# Editorial: Women in science: Public health education and promotion 2021

**DOI:** 10.3389/fpubh.2022.1011133

**Published:** 2022-09-07

**Authors:** Rosemary M. Caron, Shazia Qasim Jamshed, Melody S. Goodman, Sunjoo Kang

**Affiliations:** ^1^Department of Health Management and Policy, Master of Public Health Program, College of Health and Human Services, University of New Hampshire, Durham, NH, United States; ^2^Faculty of Pharmacy, Universiti Sultan Zainal Abidin, Kuala Lumpur, Terengganu, Malaysia; ^3^Faculty of Pharmacy, Jinnah University for Women, Karachi, Pakistan; ^4^Department of Biostatistics, School of Global Public Health, New York University, New York, NY, United States; ^5^Department of Global Health, Yonsei University Graduate School of Public Health, Seoul, South Korea

**Keywords:** women in science, public health, education, health promotion, STEMM, gender gap

Although progress in the representation of women in science, technology, engineering, mathematics, and medicine (STEMM) is slow to improve, the number of women holding STEMM faculty positions has remained stagnant or declined (1, 2). In Italy and Norway, for example, females are underrepresented in top research positions despite performing as well as their male counterparts (3). Gender inequality in scientific careers has been demonstrated by differences in productivity, citation, and salary metrics (4). Further, a disparity exists with fewer women authors from wealthy countries (i.e., Germany, Switzerland, Japan) compared to poorer countries (i.e., Serbia, Romania, Bosnia Herzegovina) (2). Potential contributing factors for these discrepancies could include prevalent gender stereotypes, absence of social capital in the form of support networks, unwelcome academic and research environments, cultural factors, and the overlap of establishing a research career with motherhood, childcare, and family obligations (1, 2). Despite improvements in the promotion of women in science, such as the National Science Foundation's ADVANCE initiative which promotes equity for faculty in the academic profession, systemic and organizational reforms in the promotion trajectory, including education, publishing, and mentoring are needed as a start to close the gender gap in academia (2, 5).

To serve as a mechanism by which to highlight the contributions of women researchers, this Research Topic features female contributions to Public Health, specifically in the field of Public Health Education and Promotion. The theme, intentionally broad in scope, sought general perspectives on a specific field of research inspired and initiated by women; articles celebrating outstanding female researchers and their contributions to public health education; and research studies led by women studying public health education and/or health promotion. To be considered for this collection of articles, the first or last author was required to identify as female. Lastly, it was recommended that early career researchers collaborate with senior female colleagues. This editorial aims to provide an overview of the key findings of the papers published in the Research Topic on Women in Science—Public Health Education and Promotion, 2021. Upon review of the articles, most research addressed maternal health issues but extended into disparate, yet contributory areas including the COVID-19 pandemic, cholera, upper respiratory infections, physical activity, and the experience of Latina women in academia. The types of articles received in response to this Research Topic were comprehensive including original research, perspectives, brief research report, study protocol, systematic review, and a community case study. The authors' work is organized and summarized according to article type below:

## Original research

Upper respiratory tract infections (URTIs) are primarily viral with many of the reported cases being bacterial. Defining and differentiating such patients is difficult because the clinical presentations connected with bacterial or viral-related URTIs commonly overlap, hence antibiotics are frequently prescribed to manage URTIs in primary care settings. Nevertheless, and other than certain exceptions, antibiotics are unnecessarily prescribed for URTIs. This frequent use of antibiotics adds a burden to healthcare systems that results in clinical failure and/or an increase in the development of antibiotic resistance. Hashmi et al. conducted a retrospective prescription analysis to document URTI-specific antibiotic prescription frequency in a public primary healthcare setting of Quetta City, Pakistan. Prescribing practices were evaluated based on the recommendations of the National Institute for Health and Care Excellence. The authors found that antibiotics were frequently prescribed for non-specific URTIs, cough, and rhinitis, even though only a limited number of patients with URTIs warranted antibiotic treatment. The authors propose developing and implementing local antibiotic guidelines and continuous medical education regarding antibiotic use. The authors also urge policymakers to introduce antimicrobial stewardship programs and guidelines in healthcare institutes.

Fang et al. conducted a cross-sectional study of the China Family Panel Studies to examine a gender phenomenon with respect to sports participation. It has been reported that women have more constraints to engage in sports than their male counterparts due to time, knowledge, and interest (6). The authors found that women who were of a high economic status and education level, unmarried, and knowledgeable about the benefits of physical activity were more likely to engage in sports than women who are overweight or are experiencing a chronic disease. In addition, women in the 50–59 age group reported having more time to engage in sports compared to younger women who are busy with attending school, family, and work. Due to the reported health benefits from engaging in physical activity (7), the authors recommend that the Chinese government develop social network policies to support sports participation, alleviate social gender norms and socio-cultural restrictions, improve transportation access to comprehensive sport facilities, and improve social media for sports engagement.

Research by Luo, Song et al. aimed to develop a college student physical literacy questionnaire (CSPLQ) to address the lack of currently available physical activity literacy assessment tools for Chinese college students. The research process of the CSPLQ consisted of two stages: item generation and validation process. Data was collected from seven Chinese colleges and universities. Evidence of content validity was provided for all processes from defining the domain, constructing definitions, generating items for expert review and response processes, content structure analysis, and relationships with other variables. The study was an initial exploratory effort aimed at designing a validated self-report questionnaire on physical activity literacy.

The theme of physical activity is further examined by Luo, Yang et al. who apply Kane's validity framework to evaluate the physical exercise peer support questionnaire (PEPSQ). The article describes the experience of using the framework and considers data related to four inferences (scoring, generalization, extrapolation, implication) that emerge from the assessment process. The findings of the study are interpreted through these inferences. The authors' findings provide evidence for the identification of peer support for physical exercise among Chinese college students. The authors conclude that the key elements that potentially affect college students' participation in physical exercise is an important part of developing health education interventions.

Ishaq et al. aimed to develop the profile and predictors of maternal Quality of Life (QoL) among pregnant women attending a primary healthcare institute in Quetta City, Pakistan. A cross-sectional study was conducted at the Obstetrics and Gynecology Department of Sandeman Provincial Hospital Quetta, Pakistan. Among the respondents, education and household income were positively associated with QoL, yet as the pregnancy progresses, there was a decrease in QoL. The authors recommend that healthcare professionals and policymakers reflect on the identified QoL factors while designing therapeutic plans and interventions for pregnant women.

Shakeel et al. conducted a cross-sectional study in Karachi, Pakistan among obstetricians and gynecologists to examine their knowledge, attitude, and practice of off-label medicine use (OLMU) for female reproductive health issues (FRHI). Although aware of OLMU, most respondents did not follow any guidelines or regulations for OLMU in their hospital due to liability and risk-to-benefit concerns for the patient. The authors recommend that tailored policies should be established to the local community context to analyze OLMU that addresses patient safety.

## Perspectives

By examining concerns about safety, compliance, and distribution through an interdisciplinary approach of public health and history, Caron and Girard Dorsey argue that historical and contemporary mistrust of immunizations which serve to challenge the successful management of a COVID-19 vaccine program in the US should be acknowledged. Unique circumstances surrounding the development of a COVID-19 vaccine, including pressure for rapid production, unclear communication from public health officials, and existing resistance to behavioral protective public health policy measures (e.g., mask-wearing) complicate widespread vaccine adoption. Public health recommendations are offered for the continued management of an effective and safe COVID-19 vaccine and necessary COVID-19 vaccine booster.

Assuring healthy populations during the COVID-19 pandemic and recognizing the role of women researchers in addressing syndemic interactions is the focus of the article by Caron and Aytur. The authors discuss the significance of a syndemic framework which examines the contributions of structural, social, economic, and environmental factors and disease interactions that synergistically act together to contribute to adverse health outcomes (8). The authors describe how the interactions among the social determinants of health (SDoH) and the COVID-19 pandemic have had different results for marginalized populations and have worsened health outcomes for many in this synergistic pandemic. The authors also discuss the role of the exposome, which is the exposure measures for an individual over their lifetime and how those exposures relate to the individual's health (9), and how this concept may help to explain why some populations experience more serious cases of COVID-19 compared to other groups. To respond to this Research Topic, Caron and Aytur also highlight, *via* specific examples, the contributions of female health professionals globally (e.g., chronic disease management, violence prevention, zoonotic transmission) to SDoH and the COVID-19 syndemic, as well as propose health policies to address syndemic-exposome interactions to help mitigate or prevent public health challenges. In particular, the authors call for transformative changes across governments, systems, and infrastructures to support gender-sensitive policies that will improve the SDoH-exposome interactions as key drivers of health.

The perspective offered by Abraido-Lanza et al. about challenges and opportunities experienced by Latina women in academia aligns well with the premise for this Research Topic. The authors analyzed data from the Association of Schools and Programs in Public Health and noted that there are <10% of instructional and tenured faculty in these member programs and schools of public health. This disparity is a form of epistemic oppression which is a type of exclusion that hinders knowledge production and advancement (10). The authors examine this claim by describing national trends on Latina representation in academia, sociopolitical contexts, family-level dynamics, gendered norms, and institutional contexts that impede the full participation of Latinas in academia. Abraido-Lanza et al. propose systemic and organizational reforms necessary to advance the career trajectories of underrepresented scholars. Representative reforms include academic administration being held accountable in establishing, implementing, and evaluating policies for the hiring, retention, and promotion of diverse staff and faculty. Structured mentoring and leadership programs for women can also provide necessary support and guidance.

The perspective offered by Sealy-Jefferson examines racial inequity between Black and white mothers, focusing on several factors accounting for the difference between the two racial groups. The perspective addresses a broad range of determinants responsible for the observed inequitable outcomes for Black people, including maternal health access and quality, maternal mortality, structural racism, mass incarceration, and poverty-stricken living environments. The author also calls for different research questions, orientations, and methodologies in studying injustices in Black maternal health. The author proposes that research questions should not rely on currently available data, or solely on the intellectual curiosity of researchers, but on relevant theories and frameworks and the use of participatory research methodologies for action.

## Brief research report

The Cooperative Extension System (Extension) is a US network, in existence for more than a century, that provides community-based education through local Extension offices affiliated with states' land grant universities. Extension education efforts have focused on youth and community development, agriculture, and family and consumer services. Washburn et al. conducted a survey based on the Consolidated Framework for Implementation Research: Intervention Characteristics, Inner Setting, Characteristics of Individuals, and Process to assess the capacity and readiness of policy, systems, and environmental (PSE) change among Family and Consumer Sciences (FCS) Extension agents in Kentucky and Tennessee. Respondents perceived PSE work as valuable while potential barriers reported included perceived complexity, readiness in reporting requirements, and a concern about taking time away from direct education efforts. The authors state that combining PSE work with traditional extension provides research-based, effective programs and interventions to improve and sustain the health and wellbeing of the communities served is necessary. The authors' findings provide informative insight for other Extension and public health organizations looking to build capacity within community-level educators to improve the population's health.

## Study protocol

The evaluation of the efficacy of preconception lifestyle interventions in preventing disease risk factors for a population of overweight and obese women in two Canadian communities, without a history of infertility, and their partners, is the aim of this study protocol developed by Hardy et al. Participants will be randomized to receive the *Healthy for my Baby* intervention or standard care in the preconception period and pregnancy. *Healthy for my Baby* is a novel behavioral intervention developed by the authors to support the adoption of healthy lifestyle habits for women and their partners in preconception and throughout pregnancy. Couples will initiate the preconception intervention by participating in a 60-min motivational interview (MI) session on healthy lifestyle habits. At the end of this session, each member will have set specific SMART (specific, measurable, achievable, relevant, time-bound) lifestyle goals in at least two of five key areas: nutrition, physical activity, sleep, wellbeing (stress, anxiety), and environment (tobacco use, drug use, alcohol consumption, or other toxic substances). Lifestyle goals will be tailored to each participant based on the priorities verbalized in the interview with the aim of improving compliance with Canadian guidelines for diet, physical activity, sleep, and alcohol consumption. Participants will then have access to a mobile phone application designed by the authors that will help them achieve three goals in different dimensions at a time through daily self-monitoring. The study hypothesis is that, compared with standard preconception and obstetrical care, this intervention will lead to an improvement in the lifestyle habits of women and their partners in the preconception period and during pregnancy and a reduction in women's weight in the interval from enrollment to 6 months of follow-up. The primary outcome of the trial is the diet quality of women, assessed with the Canadian Healthy Eating Index (C-HEI) during the preconception period, assessed at baseline, 2-, 4- and 6-months following study enrollment. The secondary objectives of the preconception period include the impact of the intervention on urinary metabolomic indicators of women's dietary exposure, the diet quality of male partners, the other lifestyle habits of women and their partners (physical activity, sleep quality, anxiety, depression, and quality of life), and anthropometric measures of women and their partners (weight, waist circumference, and body fat percentage). After completion of the primary outcome assessment, women and their partners who have achieved a pregnancy within 12 months following enrollment will be followed until the end of the pregnancy. This innovative study will be the first to document the effectiveness of an intervention combining motivational interviews and technology support to improve the lifestyle habits of women with overweight or obesity, without a history of infertility, in the preconception period. Furthermore, it will be the first study to include male or female partners in the evaluation of a preconception intervention.

## Systematic review

High rates of maternal mortality due to common preventable causes such as hemorrhage, eclampsia, and sepsis call for safe procedures like Cesarean Section (CS). Although, theoretically, the procedure is intended to protect against adverse maternal outcomes, the increase in cesarean rates in low- and middle- income countries has not been associated with improved perinatal outcomes. In addition to increased risk of neonatal and perinatal mortality in vaginal birth after cesarean (VBAC), previous CS has been reported as being associated with adverse outcomes of subsequent pregnancies such as maternal mortality, blood transfusion, admission in critical care, and hysterectomy. In 2014, the WHO proposed Robson classification for assessing, monitoring, and comparing CS rates within and between healthcare facilities over time. Jamshed et al. meta-analysis assessed women globally according to Robson's classification and reported pooled evidence on the impacts of previous CS on outcomes of subsequent pregnancy. Previous CS was found to be associated with adverse maternal outcomes in subsequent pregnancy and childbirth. The odds of experiencing adverse outcomes for women who experienced repeat-CS was higher than someone who delivered VBAC. The authors found that women who showed pre-eclampsia outcomes were three times more likely to experience repeat-CS than those who delivered VBAC. Women giving birth to their fourth child through CS can be three times more likely to experience pre-eclampsia compared to gravida. This study also revealed that women who have uterine dehiscence as an outcome are more likely to experience repeat-CS than those who delivered VBAC. There were no distinctions in the results between the repeated CS and the VBAC for preterm delivery, heavy bleeding, retained placenta, and maternal death. The authors conclude that the benefits CS can bring to reduce maternal mortality and perinatal outcomes have yet to be realized in low- and middle- income countries.

## Community case study

Cholera remains a significant public health problem, especially in Ethiopia, where vulnerable populations live in many resource-limited settings with poor access to safe and clean water and inadequate hygiene practice. Park et al. present a cholera control plan for Ethiopia, including case detection and reporting, outbreak declaration, case management, and transmission control. Specifically, the authors describe the responsibilities of healthcare facilities and various government levels for a cholera outbreak including the response and control efforts [e.g., oral cholera vaccine (OCV)]. There is a multi-year national commitment to control cholera in Ethiopia despite significant disparity in healthcare service utilization and health outcomes among people at different geographical areas and socio-economic levels. The authors propose recommendations to overcome existing challenges including improving disease surveillance, diagnostics capacity, health information reporting system, effective OCV intervention strategy, community engagement for early case detection and proper case management, and the promotion of water, sanitation, and hygiene is critical. In addition, enhanced surveillance and a comprehensive reporting system will allow the government to assess and evaluate the impact and effectiveness of vaccination when OCVs are pre-emptively used in cholera endemic hotspots or reactively in cholera outbreak settings. Lastly, the authors propose that to better estimate disease incidence, prevalence, mortality rates, and vaccination coverage rates, a multi-sector system is necessary.

## Conclusion

The research highlighted herein, addresses this specific Research Topic to demonstrate the many contributions women are making in the field of public health education and promotion globally. General perspectives on a specific field of research inspired and initiated by women; articles celebrating outstanding female researchers and their contributions to public health education; and research studies led by women studying public health education and/or health promotion comprise the Women in Public Health Education and Promotion, 2021, collection.

## Author contributions

RC led the planning and writing of the editorial. All authors contributed to the writing and review process for the editorial.

## Conflict of interest

The authors declare that the research was conducted in the absence of any commercial or financial relationships that could be construed as a potential conflict of interest.

## Publisher's note

All claims expressed in this article are solely those of the authors and do not necessarily represent those of their affiliated organizations, or those of the publisher, the editors and the reviewers. Any product that may be evaluated in this article, or claim that may be made by its manufacturer, is not guaranteed or endorsed by the publisher.
